# Challenges in Enzymatic Route of Mannitol Production

**DOI:** 10.5402/2013/914187

**Published:** 2012-12-26

**Authors:** Sheelendra Mangal Bhatt, Anand Mohan, Suresh Kumar Srivastava

**Affiliations:** ^1^Biotechnology Department, Lovely Professional University, Punjab 144 401, India; ^2^Institute of Technology, Banaras Hindu University, Varanasi 221 005, India

## Abstract

Mannitol is an important biochemical often used as medicine and in food sector, yet its biotechnological is not preffered in Industry for large scale production, which may be due to the multistep mechanism involved in hydrogenation and reduction. This paper is a comparative preview covering present chemical and biotechnological approaches existing today for mannitol production at industrial scale. Biotechnological routes are suitable for adaptation at industrial level for mannitol production, and whatever concerns are there had been discussed in detail, namely, raw materials, broad range of enzymes with high activity at elevated temperature suitable for use in reactor, cofactor limitation, reduced by-product formation, end product inhibition, and reduced utilization of mannitol for enhancing the yield with maximum volumetric productivity.

## 1. Introduction

Expanding applications in food and medical sector raises the demand of mannitol constantly, and according to an estimate, the present global market of mannitol is around $100 million with a growth rate of 5%-6% annually (2005–2009). According to Aldrich catalogue 2008, mannitol price was around $79.16 per kg [1], and current price of mannitol according to Sigma catalogues is around 42$ per Kg (2011, M4125, D-mannitol 98% pure). According to the research report entitled “Polyols: A Global Strategic Business Report” announced by Global Industry Analysts Inc., the global market for polyols has been forecasted to reach 4.0 billion pounds by the year 2015.

Around 50,000 tons/year of mannitol are produced currently by the chemical hydrogenation alone around the world via hydrogenation of 50% fructose/50% glucose syrup at high pressures and temperatures using a Raney nickel catalyst, but the major product obtained is racemic mixture of mannitol and sorbitol; *β*-fructose is hydrogenated into mannitol, whereas *α*-fructose is hydrogenated into sorbitol [2]. Substrate rich in high fructose content or pure fructose is more suitable for mannitol 50% (w/w) production via chemical hydrogenation step and is patented [3]. However, using these raw materials is very expensive, and therefore, an alternative cheap substrate based on enzymatic conversion method was used. In nutshell, chemical route has low yield 17% (w/w), requires costly operations, and produces racemic mixture, and thus, alternative biotechnological methods via microbes may be better solutions for industrial applications [4]. 

Various alternative substrates have been used by researchers for mannitol production such as pure D-glucose and its epimerization product (mannose), but this also becomes a noneconomical process pertaining to high prices of mannose. Therefore, nonepimerized glucose was used to further cheapen the cost, which is enzymatically isomerized (by glucose isomerase) to D-fructose and can be hydrogenated into mannitol in four steps, but again it was very costly [4]. Thus, the multistep approach was adapted: first by enzymatic process (mannose isomerase and glucose isomerase) and then via chemical hydrogenation, which yields high purity mannitol, but commercial availability of such enzyme cannot be feasible at all times, which again becomes a big limitation. 

Thus, the biotechnological approach has been worked out via rerouting the metabolic pathway, namely, metabolic engineering approaches utilizing suitable microbes with the possibility of minimizing these entire complex routes into single step, yielding pure mannitol using economical substrate and enzymatic hydrogenation alternative to chemical way. Nowadays, scaling up of bioreactor is a major issue, which has been worked out in detail to focus on industrial compatibility. There have been few review articles published [1, 4–6] focusing on various strategies of mannitol production but little has been discussed on scaling up problems such as media design, role of pH, role of cofermentation, and Km value of various enzymes in mannitol formation. In the present paper these issues have been reviewed which may give better insight in manipulating the production strategies for further research and adapting enzymatic methods alternative to chemical route for many industries. 

## 2. Properties and Commercial Applications

D-Mannitol was first isolated from exudates produced by the manna ash tree *Fraxinus ornus *in 1920, and as a result it is also known as mannite or mana sugar. It is a well-known low-calorie sweetener due to its half relative sweetness as compared to sucrose and has low solubility in water (around 18% w/v) compared to other isomer, and thus, its separation is easier from sorbitol after crystallization. Due to the low enthalpy around −28.9 cal/g or calorific value of 1.6 kcal/g of mannitol, it plays an important role in combating obesity due to its sensitivity to insulin, and it also gives cooling effect due to its positive enthalpies [7]. Beside being a food additive, mannitol is also used in various therapies namely, colon cancer owing to the release of short-chain fatty acid such as butyrate in colon as osmo-inhaler (in dry form) in cystic fibrosis patient owing to withdraw water into lungs that makes sticky viscous mucus thin layer giving instant relief to patient via coughing out the mucus easily during physiotherapy. In acute glaucoma, it have been used to dehydrate the vitreous humor and thus lowers the intraocular pressure (20% solution). Mannitol also find its application in making illicit drugs or as adulterants for heroin, methamphetamines. Mannitol is also commonly used in the intensive care unit; also in cerebral oedema and acute renal failure for assessment of renal function [1, 8].

## 3. Chemical Routes of Mannitol Production

Chemical methods basically rely on substrate hydrogenation into mannitol. Currently, mannitol is produced by chemical hydrogenation of fructose in presence of Raney nickel (Ni metal 2 mg/kg) at high temperature and pressure (100°C–150°C and 100°–150 lb) [6]. But it results in formation of racemic products mannitol and sorbitol. With HFCS (high fructose corn syrup) as substrate, glucose hydrogenated exclusively to sorbitol, while fructose is converted to mannitol. The use of pure fructose after separation yields pure mannitol (48%–50%) [2, 3], while invert sugar (glucose fructose 1 : 1), HFCS (containing high fructose) yield mannitol between 31% and 55% w/w. Now a days more selective bimetallic amorphous nanocatalysts made up of CoNiB (PVP-stabilized CoNiB) have been utilized for reduction of fructose and invert sugar (fructose/glucose mixture) [9, 10]. The major problems encountered during chemical hydrogenation are the use of high temperature and pressure, longer reaction time to get final product, lack of pure raw materials, leaching of metal catalyst in product, formation of recemic product and easy microbiological infections. However, Biotechnological production has also met specific problems faced in industry, therefore has not currently in practice. The major problems encountered, it is low yield of mannitol in bioreactor, low productivity, are byproduct formation such as Glycerol, Ribitol, and Ethanol, which further can act as inhibitor of various enzymes involved in mannitol production—for example, ethanol inhibits *C. magnoliae* MDH, a key enzyme for the conversion of fructose to mannitol.

## 4. Enzymatic Conversion Followed by Chemical Hydrogenations

This dual strategy had improved both the yield as well as the volumetric productivity of mannitol, since enzymatic processing of various starches (wheat, tapioca, and rice) yields HFCS (fructose) which can be chemically hydrogenated to mannitol. Enzymes used are mostly glucose isomerase owing to its optimum working condition at high temperature and pH [10]. Alternatively, mannose was used, which yields mannitol up to 36% w/w, but owing to its unavailability at large scale, it is difficult to operate at all places, and thus, D-fructose was used instead of mannose, which improves mannitol yield up to 50%–70% w/w. However, due to the reversible conversion of mannitol, it is not a preferred substrate in industries [10]. Alternatively fructose syrup (<15% glucose) as a substrate again does not have practical viability for commercialization owing to (1) unavailability of mannose isomerase and (2) since fructose-rich fractions have to be recycled several times to get the mannitol [3].

### 4.1. Biocatalytic Hydrogenation Methods

**Figure 1:**
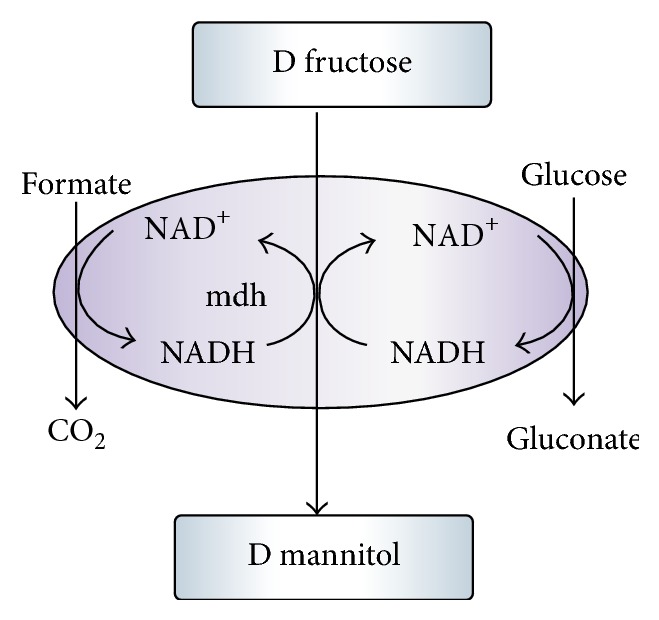
Cofactor regeneration strategies in mannitol production.

The chief enzyme accounted for enzymatic hydrogenation of fructose into mannitol is NADPH-dependent mannitol dehydrogenase (mdh) (EC 1.1.1.67), but due to unavailability of a suitable regeneration method of NAD or NAD(P)H cofactor, again it was not employed practically. Therefore, a new strategys was adapted to regenerate the cofactor which was co-fermentation of the two substrates glucose and fructose (1 : 1), where glucose gets converted into gluconate yielding NADH by glucose dehydrogenase Howaldt et al. [11] (Figure 1). 

Another strategy that has been worked out was cell biotransformation approach in resting *B. megaterium *cell where *mdh *from *Leu. pseudomesenteroides ATCC12291, *and* fdh *from (encoding formate dehydrogenase) *M. vaccae *N10 have been overexpressed and thus, NADH was regenerated successfully via the oxidation of formate to carbon dioxide and thus mannitol production increased tremendously around 10.60 g/L in the shake flask. Employing fed-batch bioreactor mannitol production was further increased up to 22.00 g/L at pH 6.0 [12]. 

A very novel technical of cofactor regeneration was adapted by some workers via electrochemical recycling of pyridine nucleotide [22], but again, retention of cofactors in the bioreactor was major obstacle; and another obstacle was the strong product inhibition of mannitol dehydrogenase, and high Km value for fructose [2]. As a result which initial fructose concentration was kept high from 100 to 140 g L^−1^ has lesser effect on mannitol productivities in *Leu. mesenteroides* ATCC 9135 [23].

## 5. Factors Affecting Mannitol Production

Beside cofactors, there are various factors that affect mannitol production such as type of microbes, substrate, cofactor NADH, enzymes, and operons, that must be discussed.

### 5.1. Type of Microorganism

Several microbes have been reported to produce mannitol. A brief account is given in Table 1.

#### 5.1.1. (a) Fungus

Major mannitol-producing fung reported are *Aspergillus, Eurotium*, [24], *Alternaria alternata, Cladosporium herbarum, Epicoccum purpurascens, Fusarium sp*. isolated from cotton dust [25], *Pencillium scabrosum* IBT JTER 4 (43 g/L mannitol), *P. aethiopicum IBT MILA* 4 (65 g/L in 150 g/L sucrose and 20 g/L yeast extract), *Aspergillus candidus* (31.0 mol%), and *P. scabrosum* (51% mole with glucose [26].)

#### 5.1.2. (b) Yeast

Two major mannitol-producing yeasts have been reported, namely, *Torulopsis versatilis and T. anomala *which produce mannitol utilizing glucose, fructose, mannose, galactose, maltose, glycerol, and xylitol to produce mannitol. *Zygosaccharomyces rouxii *can yield mannitol up to 51 mol% mannitol (0.68 gL^−1^ h^−1^) [27] while *Rhodotorula minuta *produces mannitol from D-aldopentoses [28].

Compared to batch culture, fed-batch culture was reported to produces more mannitol, that is, resting cells of *Candida magnoliae *which produces mannitol up to 67 g/L mannitol from 150 g/L fructose; while in fed-batch culture, mannitol produced was 209 g/L using fructose 250 g/L with productivity 1.03. Again, cofermentation of glucose and fructose (1 : 5) produces high concentration of mannitol up to 213 g/L [29]. 

#### 5.1.3. (c) Bacteria

Among several bacteria, the major mannitol producers reported were Lactic acid bacteria (LAB), namely, *Lactococcus, Lactobacillus, Streptococcus, Leuconostoc, Pediococcus, Aerococcus, Carnobacterium, Enterococcus, Oenococcus, Tetragenococcus, Vagococcus* and *Weisella* [30]. But out of 80 species of Lactobacillus (L.) *L. sanfranciscensis* reported to produce better yield of mannitol [31]. Recently some of Leuconostoc (Lue.) species reported to produce mannitol >80% w/v from fructose (50–100 g/L) at high temperature, high pH, and at high osmotic tolerance in batch mode without substrate inhibition [15]. 

 Therefore, as compared to fungal species; bacteria reported to have better production times (Leuconostoc species), no substrate inhibition and better productivity. Some of the mannitol-producing LABs are enlisted in Table 3. 

### 5.2. LAB Fermentation Pathway

**Figure 2:**
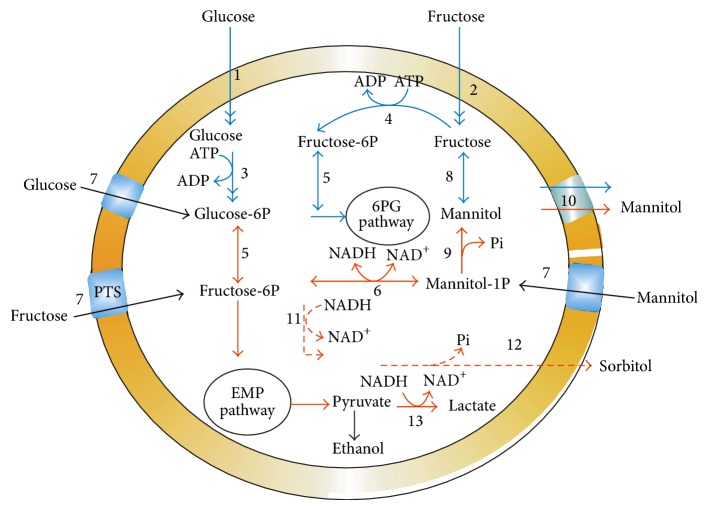
Mannitol production pathways: double arrow (→→) heterofermentative and single arrow homofermentative (→) LAB while sorbitol production pathway shown by broken arrow (→) is. Enzymes are numbered, (1) glucose permease, (2) fructose permease, (3) glucokinase, (4) fructokinase, (5) phosphoglucose isomerase, (6) mannitol-1-phosphate dehydrogenase, (7) PEP dependentPTS, (8) mannitol dehydrogenase, (9) mannitol-1-phosphatase, (10) unspecified hexitol transport, (11) sorbitol -6-phosphate dehydrogenase, (12) unspecified, (13) lactate dehydrogenase, PTS, phosphotransferase system; 6PG, 6-phosphaogluconate; EMP, embeden-Meyerhhof-parnas reaction 9 + 10 and 12 may occur via PTS.

The LAB utilize either of the two main hexose fermentation pathways, (1)-homolactic fermentation (**EMP** pathway) and (2)-heterolactic fermentation (6-phosphogluconate/phosphoketolase (6-PG/PK) pathway). Further, the obligate homofermentative LAB can only ferment sugars by glycolysis, while the obligate heterofermentative LAB uses only the 6-PG/PK pathwayh while the facultative heterofermentative LAB have the capability to utilize both pathways. In the 6-phosphogluconate/phosphoketolase (6-PG/PK) pathway, lactic acid is not the only end product; in addition, CO_2_ and ethanol are also produced (Figure 2).

Therefore, heterofermentative LAB can form mannitol using fructose broth as an electron acceptor and a growth substrate (Figure 2, (1)). Part of the fructose is reduced to mannitol by mannitol dehydrogenase and part is phosphorylated by fructokinase and isomerizes to glucose-6-P, which is fermented normally in the 6-PG/PK pathway. The net equation of fructose fermentation may be depicted as

(1) 3  fructose+2 ADP+2 Pi  →mdhlactate+acetate+CO2+2 mannitol+2 ATP

Mannitol produced by LAB uses an enzyme said as *mdh,* which is present inside the cell and reduces D-fructose to D-mannitol in presence of NAD(P)H cofactor (dependent mannitol dehydrogenase EC 1.1.1.67) as shown

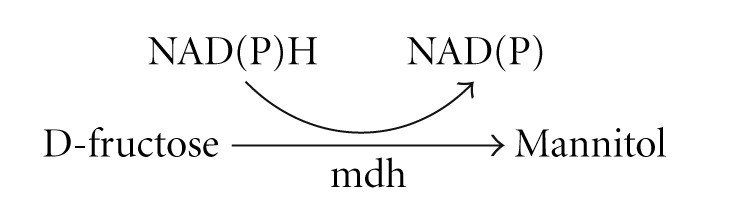
(2)


This NAD(P)H unavailability sometimes poses a strong product inhibition to *mdh* enzyme, namely, presence of oxygen reoxidises the NADH by NOX (NADH oxidase) into water or hydrogen peroxide in several LAB and thus they become unavailable [2]. Von et al. [23] also reported that Low pH and anaerobic condition favors an increases ratio of NADH/NAD^+^ while ethanol, increases this ratio more, suggesting the role of cofactor limitations and electron accepter during fermentation. Opposite to this *L. delbrueckii *ethanol production greatly hampers, if NADH supply was restored externally in presence of glucose suggesting role of NADH in enzyme activation or deactivations [32]. This also proved that an optimum pH range of 6-7 maintains a high NADH/NAD^+^ ratio essential for increasing the glycolytic flux but low pH may divert this flux and may inhibit pyruvate dehydrogenase [33].

### 5.3. Alternative Raw Material

Beside fructose, some pentose sugars, namely, ribose, xylose, arabinose, and lyxose, have been well documented to be utilized by *Rhodotorula minuta;* glycerol by *Candida *[34]; starch, namely, maize corn, wheat, and tapioca; beside this cashew apple juice, Inulin well utilized by several lactobacillus [6], raw milk, fermented milk, cereal foods fruits vegetables and sugar factory syrup by various microbes [15, 34].

Sucrose (molasses) which is an cheaper option have been reported to produce mannitol after subsequent hydrolysis into invert sugar (50% fructose and 50% glucose), and after purifying fructose to 90%–95% by chromatography procedures a high yield of mannitol have been reported, and thus, 4 tons of sugar yielded 1 ton mannitol [35]. Cofermentation of Inuline along with the fructose (3 : 5) in SSF mode reported to work well (simultaneous saccharification and fermentation) at low pH 5.0 and 37°C, and thus, mannitol obtained was 400 g/L mannitol [4].

 Mass balance equation of sucrose and fructose (1 : 1) cofermentation has been depicted

(3) Sucrose+Fructose→2 Fructose+Glucose→2 Mannitol+Lactic  acid  +Acetic  acid+CO2

Alternatively, replacement of fructose with molasses in low-cost nitrogen, CSL (fructose: CSL, 1 : 1) resulted in mannitol yielding up to 150 g/L in 16 h in continuous fed-batch process as well as (40 g/L/h) membrane cycle reactor by *L. intermedius *NRRL B-3693, *Leu. mesenteroides sub sp dextranicum, L. cellobiosus, L. fermentum, L. buchneri, L. brevis *and *L. citrovorum*. Thus, economical raw material such as high fructose syrup and corn steep liquor in place of expensive yeast extract, with protein hydrolysate, may be a better option at Industrial scale for mannitol production [6].

### 5.4. Mechanism of Cofermentation

Cofermentation of two substrates is reported to be stimulatory for certain enzymes (dehydrogenase gene expressions); as a result more availability of electron acceptor such as NADH is possible; which is often limited during mannitol production. Therefore, cofermentation of both glucose and fructose, glucose is used as an energy source while fructose is used as an electron acceptor [30]. Similarly, Maltose and glucose cofermentation results in the CO_2_, lactate and ethanol formation, while external supply of electron acceptors, (fructose, citrate, fumarate, or malate) resulted in ATP and acetate instead of ethanol formation in some heterofermentative Lactobacilli, suggesting that metabolism can be diverted to other pathway and final product may depends upon the kind of substrate supplied [31]. Another explanation behind this working strategy may be due to high theoretical yield of mannitol from minimum initial sugar 66.7 mol% which is high compared to fructose alone as shown in (1) and (4), where theoretically, fructose and glucose both should yield high mannitol as compared to fructose alone (3); in some cases it has also been reported that after glucose depletion, mannitol production ceases at substantially low amount due to activation of mannitol utilization pathway [36, 37].

(4) 2 Fructose+Glucose→2 Mannitol+Lactic  acid  +Acetic  acid+CO2

Therefore, same strategies have reported to work well with glucose and glycerol cofermentation by *L. brevis *[30], or with maltose and glycerol in *L. reuteri *[38] or glucose and citrate (to increase diacetyl production) by *L. mesenteroides *subspecies *cremoris, lactococcus lactis* and *O. oeni *[39].

### 5.5. Redox Balance Strategies

In mannitol production overall redox balance is maintained by LDH enzyme through pyruvate reduction which regenerates NAD^+^ and the same is consumed during glycolysis in most of the LAB pathway; Only under certain specific conditions, LAB uses alternative electron acceptors to regenerate NAD^+^ as has been observed in some heterofermentative LAB that utilizes part of fructose as electron acceptor [5] Figure 2. This is the reason why only one third of fructose is reported to be utilized by phosphoketolase pathway while two third were utilized for reoxidation of NAD(P)H (2) with *L. lactis*, when effect of PK and LDH activity was observed after glucose depletion [18]. Therefore, *ldh *mutant of *L. lactis *was reported to have increased regeneration of NAD^+^ via alternative pathway [5, 19]. In experiment with *Enterococcus faecalis *NADH/NAD^+^ ratio was determined [36] at high pH where maximum amount of this ratio was maintained with mannitol catabolism. Therefore, highest yield of mannitol (85 mol-%) has been reported at low pH (4-5) and high temperature (20°–30°C) [6]. In another report of Neves et al. [18], nongrowing LDH-deficient cell of *L. lactis *reported to produce a high amount of mannitol as compared to growing cells. The great difference was owing to more accumulation of NADH in the resting cells. This is obvious since in the resting cells no ATP is requisite for biomass production, thus, ATP demand was low [40], Consequently, ATP-generating steps become less important, thus, more yield of mannitol resulted. In these cases, mannitol production was via an alternative pathway to regenerate NAD^+^ instead of lactate formation (Bhatt and Srivastava [32]).

### 5.6. Role of Mannitol Dehydrogenase

In most of the LAB, mannitol dehydrogenase plays an important role in the reduction of fructose to mannitol with concomitant oxidation of NADH to NADP, therefore, most of the MDH is NADPH-dependent and belongs to a family of Zn^2+^-independent long-chain alcohol dehydrogenase which catalyzes the regiospecific NAD^+^-dependent oxidation. From Brenda and KEGG database, the following types of dehydrogenase were reported to occur.

- Mannitol-1-phosphate 5-dehydrogenase (EC 1.1.1.17) (gene mtlD), which catalyzes the NAD-dependent reduction of mannitol 1-phosphate into fructose 6-phosphate or reverse reaction.
- Mannitol 2-dehydrogenase (EC 1.1.1.67) (gene mtlK), which catalyzes the NAD-dependent reduction of mannitol into fructose.
- Mannonate oxidoreductase (EC 1.1.1.57) (fructuronate reductase) (gene uxuB), which catalyzes the NAD-dependent reduction of fructuronate into mannonate.
- The NADPH-dependent MDH (EC 1.1.1.138).
- *Escherichia coli* hypothetical protein *ydfI*.
- *Escherichia coli* hypothetical protein *yeiQ*.
- Yeast hypothetical protein *YEL070w*.

Thus, both NADH- and NADPH-dependent MDHs have been purified from a number of microorganisms, namely, *L. brevis, Leu. mesenteroides, P. fluorescens, Rhodobacter spaeroides, S. cerevisiae, *and *Toluspora delbrueckii *[41, 42], and also from several fungi such as *A. parasiticus, C. magnoliae, Z. mobilis, and Gluconobacter suboxydans *[29, 43, 44].

MDH isolated from *Pseudomonas fluorescens *shows that the D-mannitol oxidation rates decreases at low pH. The purified MDH of *L. sanfranciscensis *reported toexhibit a narrow substrate spectrum which is much beneficial from other broad range MDH enzymes because of utilization of fructose only, among various substrates selected such as glucose, arabinose, xylose or mannose. The fructose reduction activity of MDH was reported to be enhanced by various ions namely NH_4_, Ca^+2^, Li, Mg^+2^, K, and EDTA, whereas Fe^+2^, Zn^+2^ and Mn^+2^ inhibits the MDH enzyme reducing fructose reduction [45]. In one report, the disruption of *ldh *resulted in *L. lactis* resulting in increased expression of mannitol 1-phosphate dehydrogenase (MPDH) and mannitol 1-phosphate phosphatase (MPase), and both were reported to be involved in the synthesis of mannitol from fructose-6-P [20]. Thus, glucose converted to mannitol-1-P, which is subsequently dephosphorylated to mannitol by MP. However, a disadvantage of the mannitol production via MPDH and MPase is that fructose 6-phosphate, is also part of the glycolytic pathway, thus creating a direct competition between mannitol production and glycolysis. Wisselink et al. [5] expressed double gene mannitol 1-phosphate dehydrogenase gene (*mtlD*) derived from *L. plantarum *in LDH-deficient *L. lactis *using nisin-inducible expression, and mannitol-1-phosphate phosphatase of *Eimeria tenella *(a protozoan parasite) resulted in 25%–50% of glucose conversion to mannitol in resting state fermentation. By introduction of a functional MDH in *L. lactis*, the mannitol production pathway was reported not to be competitive with glycolysis.

Gaspar et al. [19] succeeded in increasing further mannitol production by constructing double mutant of *L. lactis *strains (by mutating the mannitol transport system of the LDH-deficient *L. lactis *strain, the *mtlA *or *mtlF *genes encoding *EIICBMtl *and *EIIAMtl*, resp.). As a result, in the resting state, these double mutant strains converted about 30% of glucose to mannitol.

Recently, mannitol has been produced from glucose using two enzymes in two steps such as Xylose isomerase for conversion of glucose into fructose, and then fructose was reduced to mannitol by MDH isolated from *Thermotoga maritima *(*TmMtDH*) (optimally active between 90 and 100°C) which has been cloned and expressed in *Escherichia coli *for industrial scale up procedure in bioreactor where temperature rises during fermentation [46].

**Figure 3:**
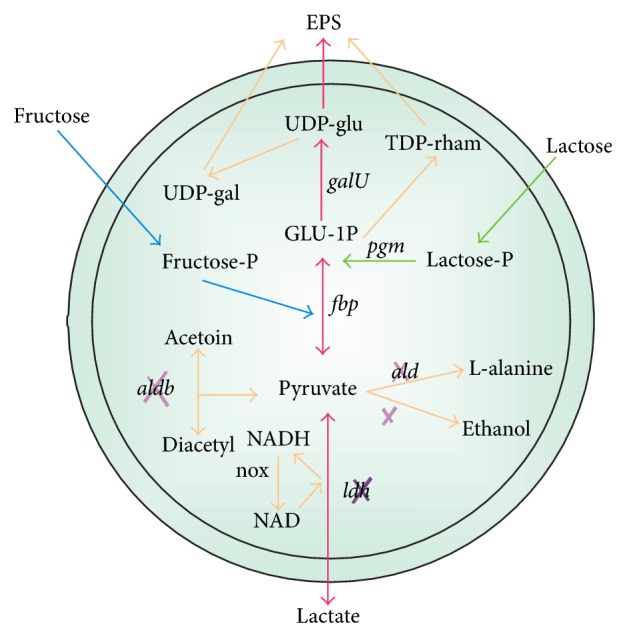
Metabolic engineering strategies in Homofermentative LAB. ALD: L alanine dehydrogenase; ALDB: alphaacetatelactate dehydrogenase; FBP: fructose bis phosphate; GAL: galactose; NOX NADH oxidase; PGM: phosphoglucomutase; rham: rhamnose.

Figure 3 depicts the various gene-engineering strategies adapted in homofermentative pathway. Thus, one of the main strategies reported to maximize the NADH regeneration was inactivating both of *ldh *gene (*ldh-L *as well as *ldh-D*) [19]. In same attempt *Pichia pastoris *formaldehyde dehydrogenase (FLD) gene was overexpressed for butanediol production [47] in *Klebsiella oxytoca *where expression of *fdh *gene from *Candida boidinii *resulted in higher intracellular concentrations of both NADH and NAD^+^ during the fermentation without affecting the NADH/NAD^+^ ratio [48]. Still more research regarding this is required to further increase the yield and volumetric productivity.

### 5.7. Role of Transporter Molecule

**Figure 4:**
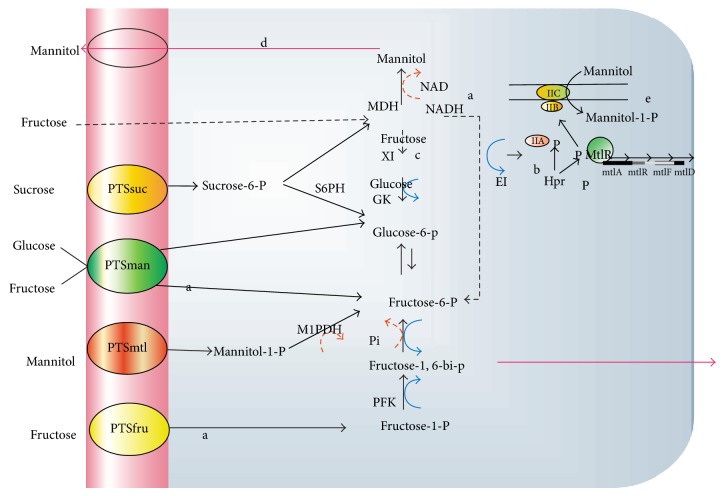
Proposed sucrose metabolism in *L. lactis *NZ9800/NZ9841 overproducer strain MDH isolated from *Leuconostoc mesenteroides*. (a), phosphorylation of fructose into fructose 6-phosphate by fructokinase (b), export and the subsequent import of fructose by the fructose PTS (PTSfru) or the mannose PTS (PTSman) (c), and the conversion of fructose into glucose by a xylose isomerase-like enzyme activity (d). Mannitol is exported by an unknown mechanism, and is possibly re-utilized by the mannitol PTS (PTSmtl) [18]. (e) Mannitol uptake mechanism. Enzyme abbreviations: sucrose 6-phosphate hydrolase (S6PH), mannitol dehydrogenase (MDH), fructokinase (FK), glucokinase (GK), 1-phosphofructokinase (1-PFK), xylose isomerase (XI), phosphotransferase system (PTS).

Figure 4 depicts the transporter molecule (PTS) having role in transport of various substrate for metabolism. In homofermentative LAB (*L. lactis*), PTSman is reported to be the main transporter for glucose and fructose, which yields fructose 6-phosphate after phosphorylation.

Details of the mechanism have been elucidated in Figure 4. Sucrose is reported to be transported by sucrose-specific PTS, resulting in the formation of sucrose 6-phosphate, which then hydrolyzed into glucose 6-phosphate and fructose. Glucose 6-phosphate is reported to enter glycolysis as such, whereas the fructose unit was phosphorylated by an ATP-dependent fructokinase [18].

**Figure 5:**
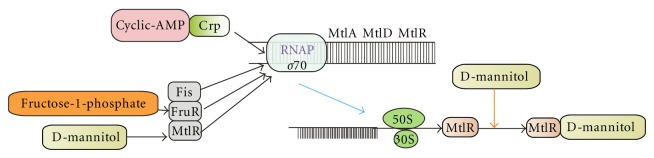
Mechanism of action of *mtlADR *operon. The transcriptional activator MtlR, which in the presence of mannitol produces derepression of the *mtlADR *operon, but when glucose is present in the medium, this operon is a target of catabolite repression (through CRP), independent of MtlR. MtlA takes up exogenous mannitol, releasing the phosphate ester, mannitol-1-P, into the cell cytoplasm, but it is not clear which of the two metabolites is the inducer of MtlR http://biocyc.org/ECOLI/NEW-IMAGE?type=OPERON&object=TU00193.

Beside this, pH is also thought to play an important role in modulation of mannitol transporter protein. In *L. lactis *there exits the mannose-PTS system (PTSman) (Figure 5) [49], which, besides glucose, is reported to transport 2-deoxy-D-glucose, mannose, glucosamine, and fructose.

A second PTS system that exhibits specificity to glucose and *α*-methyl-glucoside is the glucose-PTS system which has also been described for some strains. Alternatively, glucose was reported to be transported via a permease and subsequently phosphorylated by an ATP-dependent glucokinase while fructose uptake was reported to be mediated by the PTSman (Thompson [49]). Maltose was thought to transported by an ATP-dependent permease, and is converted to glucose and *β*-glucose 1-phosphate via the action of a Pi-dependent maltose phosphorylase [50]. To improve the efflux of mannitol, Costenoble et al. [51], created a glycerol defective mutant of *Saccharomyces cerevisiae *expressing the mtlD gene from *E. coli *coding for NADH-dependent M-1-P dehydrogenase for mannitol production under anaerobic conditions. There was an unusual mdh detected in *L. brevis *similar to *L. fermentum *and *L. sanfranciscensis *which can utilize both NADH and NADPH as cofactor while in eukaryotic fungi most of the mdh is NADPH-dependent [6, 16].

Thus it can be concluded that compare to other microbes LAB were capable of utilizing various type of substrate and thus can be suitably engineered for overproduction of mannitol as well as for efflux of mannitol.

### 5.8. Recombinant Overproducer

Lists of some recombinant overproducer strains have been tabulated in Table 2. Many recombinant strain of *E. coli *have been engineered and called overproducer owing to the enhanced capability of mannitol production. Beside this, several genetic modifications have been done to increase the fermentation rate. Genes reported to be targeted were *ldh*, *mdh, fdh, *and *adh, *and some *pts *that helped to reroute the central carbon metabolism towards mannitol formation.

A recombinant strain of *E. coli *for *mdh *isolated from *P. fluorescens *DSM 50106 [54] has been created, which was having high regeneration capacity of NADH (due to formate dehydrogenase-mediated oxidation of formate into CO_2_) along with high turnover numbers (approximately 1000 for a single round of D-fructose conversion) and D-mannitol productivity reported was 2.25 g/(L h) with mannitol production 72 g/L (80% of fructose was converted into D-Mannitol). An LDH-deficient strain was reported with reduced phosphofructokinase (*pfk*) activity and overexpressed mtlD from *L. plantarum in Lue. lactis *has been reported by Wisselink et al. [5]. Kaup et al. [53] constructed an *Escherichia coli *strain for NAD^+^-dependent MDH isolated from *Leu. Pseudomesenteroides *ATCC 12291 and NAD^+^-dependent formate dehydrogenase (FDH) from *Mycobacterium vaccae *N10 for NADH regeneration, with the glucose facilitator gene from *Zymomonas mobilis *for the uptake of fructose without concomitant phosphorylation. Thus, around 66 g mannitol was produced from 90 g fructose L^−1^ within 8 h with a yield of 73% and a specific mannitol productivity of >4 g per g cell dry weight (cdw) h^−1^. Kaup et al. [53] further reported that supplementation of extracellular GI in *E. coli *strain resulted in the formation of 145.6 g mannitol from 180 g glucose L^−1^ due to coexpression of the *xylA *gene of *E. coli *in this recombinant strain which formed 83.7 g mannitol from 180 g glucose L^−1^. In resting cells of *E. coli *BL21 (DE3) three genes were simultaneously expressed such as *mdh, fdh *and *glf *which encodes mannitol, formate dehydrogenase and a sugar facilitator, respectively, and thus the productivity of d-mannitol formation obtained with the strain expressing the additional *fupL *gene was enhanced by 20%. In an expression system a fusion protein was prepared for mannitol-2-dehydrogenase mtlK in pRSET vector in *E. coli *BL21pLysS on isopropyl-~-D-thiogalactopyranoside induction system for mannitol formation at pH 5.35 [60]. Similarly genetically engineered *L. plantarum *TF103 carrying the mtlK gene of *L. brevis *overexpressor reported to produce mannitol from glucose. Liu et al. [45] and Bäumchen and Bringer-Meyer [12] reported an overexpressor strain of *C. glutamicum *for MDH gene from *Leu. pseudomesenteroides *and coexpressed FDH gene (*fdh*) from *Corynebacterium glutamicum *ATCC 13032. As a result mannitol production rate observed was 0.22 g (g cdw)L^−1^ h^−1^. Expression of the glucose/fructose facilitator gene (*glf*) from *Z. mobilis *further increased productivity of mannitol to 1.25 g/g cdw/Lh^−1^, yielding 87 g mannitol from 93.7 g fructose while in repetitive fed-batch mode resulted in production of 285 g mannitol in 96 h with an average productivity of 1.0 g (g cdw) L^−1^ h^−1^. 

### 5.9. Operon Involved in Mannitol Metabolism

The mannitol operon has been worked out in detail and is reported to be conserved and consists of *MtlR, MtlA*, and *MtlD *also called as mtlADR operon. Many Gram-negative bacterial families, such as *Shigella *[61], *Salmonella *[62], *Yersinia *[63], *Klebsiella *[64], and *Vibrio *[65] reported to have mannitol operon with minor difference in their PTS system. In Figure 5. *MtlR *is a transcriptional activator while*, MtlA *takes up exogenous mannitol and *MtlD *is reported to involve in the conversion of fructose to mannitol. The uptake system comprises several genes encoded in the single operon (*mtlA, mtlR, and mtlF*) [65, 66].

Similar gene has been reported in Gram-negative bacteria named as *yggD, *encoding a sequence homolog of *MtlR, *but was not clustered with *mtlA, *and *mtlD *as *mtlR *as reported in a typical mannitol operon. Its gene neighbors vary considerably among organisms, even among strains, and its position provides little insight into its function (Figure 5).

It has been reported that in certain condition the *mtlD *gene was not expressed in *B. stearothermophilus. *As a result no mannitol production took place which was due to expression of mannitol operon repressor (MtlR) gene. Figure 5. In *B. stearothermophilus *MtlR was composed of 697 amino acids and contains a helix-turn-helix DNA-binding motif with two anti-terminators-like PTS regulatory domains [67]. Its obvious role has been elucidated out and found to be regulated mainly by phosphorylation which decides that mannitol has to be secreted or must be taken up. Still some understanding is required regarding its exogenous control where cell can be manipulated to secrete the cells.

### 5.10. Km Improvements for Mannitol Production

**Figure 6:**
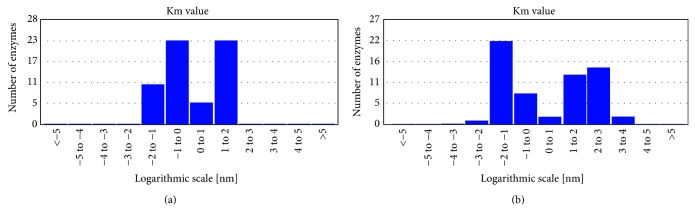
(a) Km Values for the enzymes 1.1.1.67 (b) Km Values for the enzymes 1.1.1.138 (source Brenda data base).

From BRENDA enzyme data base server, a plot (Figure 6) was obtained between Km versus number of bacteria shows range of Km and also has been depicted in Table 3 varying from 0.44 to 71 mm for fructose [52] and for mannitol from 0.29 to 78 mm [16, 52, 63]. From database following type of MDH reported to exist (1) Mannitol 1-phosphate dehydrogenase EC 1.1.1.17 (mtlD); (2) The NADPH-dependent MDH (EC 1.1.1.138) and (3) EC 1.1.1.67, encoded by mtlK from *L. intermedius *(NRRL B-3693). Most of the enzyme was having the higher Km for fructose which is the main cause of lower production of mannitol [5].

 Some LAB reported to have low km as in *L. intermedius *NRRL B-3693 where Km for fructose to mannitol was 20 mm at pH 5.0 while mannitol to fructose was higher at pH 7.0 [68]. For *L. sanfranciscensis *the apparent Km for fructose was 24 mm fructose while for mannitol oxidation step reported was 78 mm mannitol [16]. The optimum pH reported for the reduction of fructose to mannitol was 5.8 and while for the oxidation of mannitol pH was 8. Similar differences in pH were reported for *Lue. mesenteroides *(for reduction, pH 5.8 and oxidation, 8.6) [52]. Several thermophilic microbes were reported to have better stability of enzyme at higher temperature and effective Km suitable for bioreactor applications such as *Thermotoga maritima *TM0298. TmMtDH from overexpressed in *E. coli *were reported to act both on fructose and mannitol at temperature range 90 and 100°C and retains 63% of its activity at 120°C while it shows no activity at room temperature. TmMtDH reported to show higher *V*_max_ with NADPH than with NADH, but its catalytic efficiency was 2.2 times higher with NADH than with NADPH and 33 times higher with NAD^+^ than with NADP^+^. This cofactor specificity was explained due to presence of negatively charged residues (Glu193, Asp195, and Glu196) downstream of the NAD(P) interaction site, the glycine motif. [63]. Yet a novel effort have been done to increase the mdh activity by tagging six histidine codons to the 3′-end of the mdh gene and final expression in *Escherichia coli M15 *[52]. Still more focus on engineering of mdh enzyme is required which can further improve the activity.

## 6. Concluding Remarks

The biological pathway for mannitol production still has not been adapted in the industry in spite of several research report published in detail. Various organisms belonging to genera LAB have been reported to produce high yield of mannitol up to 227 g/L utilizing economical substrate such as starch, HFCS or fructose and inuline which seems suitable for industrial purposes. The genetic engineering of *E. coli *strains with *mdh *gene of *T. maritima *may prove to be boon to bioreactor design to cope with high temperature and pH rise during fermentation. Still some investigations are required regarding improvement of enzyme activity. But certainly cofermentation strategies has reported to work well in all the cases.

## Figures and Tables

**Table 1:** LAB used for mannitol production with their yield and their carbon source.

Lactic acid bacteria	Carbon source	Fermentation condition	Yield/Productivity	References
Leuconostoc* pseudomesenteroides *	Fructose	pH 4.5	6.3 g/L/h 94 mol%	[2]
*ATCC 12291* *Leuc. pseudomesenteroides (immobilized) *	Fructose	pH 4.5 Temp 20°C	1 g/L/h 85 mol%	[2]
*Leuc. pseudomesenteroides *	On hydrolyzed starch (fructose and glucose)	Batch culture	3.8 g/L/h 92 mol%	[2]
*Leuc. pseudomesenteroides *	(Fructose and glucose)	Fed-batch culture	6.3 g/L/h 85 mol%	[2]
*Leuc. pseudomesenteroides (mutant) immobilized on polyurethane foam *	Hydrolyzed starch	Fed-batch culture	7.7 g/L/h 85 mol%	[2]
*Leuc. pseudomesenteroides (mutant) immobilized on polyurethane foam *	Hydrolyzed starch	Continuous mode	8.9 g/L/h	
*Oenococcus oeni *	Glucose and fructose	Batch culture normal temp Ph	0.2 g/L/h 83 mol%	[13]
*Lactobacillus sp*. *(L.) *	Fructose	Flask culture	0.72 mol/mol fructose <1 g/L/h 86 mol%	[14]
*Leuconostoc mesenteroides *	Fructose	Flask culture	>80%	[15]
*L. sanfranciscensis *	Fructose glucose	Fed-batch	0.5 g/L/h	[16]
*Leuc. pseudomesenteroides* *ATCC 12291 (immobilized cell) *	Fructose glucose	Batch	30 g/L/h 85 mol%	[17]
*Lactococcus lactis strain F17851 (nongrowing) *	Glucose	Batch	90 mM	[18]
*F110089 ldh mutant *	Glucose	Batch	5.5 g/L/h	[19]
*F19630 double mutant *	Glucose	Batch	5.7 g/L/h	[19]
*Nongrowing MG1363 *	Glucose	Batch	18.3 g/L/h	[20]
*Non growing MG1363 *	Glucose	Batch	32.8 mol of product/mol of glucose	[19]
*L*. *intermedius NRRL B-3693 *	Fructose	pH 5.0 temp 37°C	64% w/w 4 g/L/h	[21]
*L*. *intermedius NRRL B-3693 *	Fructose	Fed batch	95% w/w 5.9 g/L/h	[21]
*L*. *intermedius NRRL B-3693 *	Fructose	Continuous recycle	40 g/L/h	[21]
*L*. *intermedius NRRL B-3693 *	Molasses and fructose syrup (1 : 1) fructose glucose (4 : 1)	Media design using soy peptone (5 g/L) corn steep liquor (50 g/L)	69% w/w 4.7 g/L/h	[6]

**Table 2:** Recombinant strains of *E*. *coli* where *mdh* genes from different microbes have been cloned for mannitol production. (source from BRENDA database).

Cloned/Commentary	Organism	Literature
MtDH is expressed in *Escherichia coli *BL21 (DE3)	*Lactobacillus reuteri *	[46]
Fusion of six His codons to the 3′ end of the mdh gene and expression in *Escherichia coli* M15. The enzyme shares significant sequence similarity with the medium-chain dehydrogenase/reductase protein family	*Leuconostoc mesenteroides *	[52]
MDH expression in *Escherichia coli*, affecting strong catalytic activity of NADH-dependent reduction of D-fructose to D-mannitol in cell extracts of the recombinant *Escherichia coli* strain	*Leuconostoc pseudomesenteroides *	[53]
Gene mdh, high level expression in *Bacillus *	*Leuconostoc pseudomesenteroides *	[12]
MDH gene subcloned into vector pDEST110 and overexpressed in different strains of *Escherichia coli* (BL21 (DE3) plysS, JM109, Origami(DE3) or M15)	*Pseudomonas fluorescens *	[54]
MDH expressed in *Escherichia coli* BL21 (DE3) cells; the TM0298 gene subcloned into the NdeI and XhoI sites of pET24a(+) to yield plasmid pTmMtDH, from which MtDH is expressed with a C-terminal His6-tag in *Escherichia coli* BL21 (DE3)	*Thermotoga maritime *	[46]
Gene mtdh, expression of the His-tagged enzyme in *Escherichia coli* strain BL21 (DE3)	*Thermotoga maritime *	[46]

**Table 3:** Km values of MDH for different substrates (source from BRENDA database).

Km value [mm]	Km value [mm] maximum	Substrate	Organism	Commentary	Literature
1.8	6.5	D-Arabinitol	*Rhodobacter sphaeroides *	—	[41]
0.44	—	D-Fructose	*Pseudomonas fluorescens *	in 50 mm glycine/NaOH buffer at pH 10	[55]
16.3	79.2	D-Fructose	*Rhodobacter sphaeroides *	—	[41]
50.97	—	D-Fructose	*Thermotoga maritima *	at 80°C and pH 6.1; at 80°C, pH 6.1	[46]
71	—	D-Fructose	*Leuconostoc mesenteroides *	pH 5.3	[52]
32	—	D-Glucitol	*Rhodobacter sphaeroides *	—	[41]
0.29	21.8	D-Mannitol	*Platymonas subcordiformis *	—	[56]
0.29	21.8	D-Mannitol	*Rhodobacter sphaeroides *	—	[41]
0.29	21.8	D-Mannitol	*Saccharomyces cerevisiae *	—	[57]
1.2	—	D-Mannitol	*Pseudomonas fluorescens *	recombinant protein	[58]
5.51	—	D-Mannitol	*Thermotoga maritima *	at 80°C and pH 6.1;	[41]
12	—	D-Mannitol	*Leuconostoc pseudomesenteroides *	pH 8.6, 30°C	[59]
13.23	—	D-Mannitol	*Thermotoga maritima *	at 60°C and pH 6.1	[41]
32	—	D-Mannitol	*Leuconostoc mesenteroides *	pH 8.6	[52]
78	—	D-Mannitol	*Lactobacillus sanfranciscensis*	25°C	[16]
